# Value of radiomics in differential diagnosis of chromophobe renal cell carcinoma and renal oncocytoma

**DOI:** 10.1007/s00261-019-02269-9

**Published:** 2019-10-29

**Authors:** Yajuan Li, Xialing Huang, Yuwei Xia, Liling Long

**Affiliations:** 1 Department of Radiology, The First Affiliated Hospital of Guangxi Medical University, No. 6 Shuangyong Road, Nanning, Guangxi China; 2 Huiying Medical Technology Co., Ltd, Room A206, B2, Dongsheng Science and Technology Park, HaiDian District, Beijing, 100192 China

**Keywords:** Renal cell carcinoma, Oncocytoma, Radiomics, Computed tomography, Machine learning, Differential diagnosis

## Abstract

**Purpose:**

To explore the value of CT-enhanced quantitative features combined with machine learning for differential diagnosis of renal chromophobe cell carcinoma (chRCC) and renal oncocytoma (RO).

**Methods:**

Sixty-one cases of renal tumors (chRCC = 44; RO = 17) that were pathologically confirmed at our hospital between 2008 and 2018 were retrospectively analyzed. All patients had undergone preoperative enhanced CT scans including the corticomedullary (CMP), nephrographic (NP), and excretory phases (EP) of contrast enhancement. Volumes of interest (VOIs), including lesions on the images, were manually delineated using the RadCloud platform. A LASSO regression algorithm was used to screen the image features extracted from all VOIs. Five machine learning classifications were trained to distinguish chRCC from RO by using a fivefold cross-validation strategy. The performance of the classifier was mainly evaluated by areas under the receiver operating characteristic (ROC) curve and accuracy.

**Results:**

In total, 1029 features were extracted from CMP, NP, and EP. The LASSO regression algorithm was used to screen out the four, four, and six best features, respectively, and eight features were selected when CMP and NP were combined. All five classifiers had good diagnostic performance, with area under the curve (AUC) values greater than 0.850, and support vector machine (SVM) classifier showed a diagnostic accuracy of 0.945 (AUC 0.964 ± 0.054; sensitivity 0.999; specificity 0.800), showing the best performance.

**Conclusions:**

Accurate preoperative differential diagnosis of chRCC and RO can be facilitated by a combination of CT-enhanced quantitative features and machine learning.

## Introduction

The incidence of renal cell carcinoma is increasing worldwide [1]. Chromophobe cell carcinoma (chRCC) of the kidney is second only to clear cell carcinoma of the kidney and papillary cell carcinoma of the kidney [1–3]. Renal oncocytoma (RO) is a benign renal tumor, accounting for about 3–7% of all renal tumors [4, 5]. Medical imaging plays an important role in the clinical management of renal tumors, such as detection of renal tumors, prediction of benign and malignant tumors, grading, and surgical treatment [6, 7]. Studies have shown that chRCC and RO not only overlap in morphological and immunological manifestations, but also have similar imaging manifestations [8, 9]. Although some researchers believe that a central scar is the characteristic of RO, its proportion is only about 33% [4, 6], but there are also a few cases of chRCC with a central scar [8]. Therefore, it is obviously impossible to distinguish the two pathological types by the presence or absence of a central scar. Some reports suggest that there are some differences in the enhancement degree of CT between the two [9]. The enhancement in chRCC is slightly higher than that in RO, but the difference in the CT value is small and is greatly influenced by subjective factors. There are also studies showing that many MR findings for chRCC and RO are quite similar, such as a central scar, segmental enhancement inversion, and enhancement characteristics of each phase, none of which can accurately identify the two [10].

At present, the differential diagnosis of benign and malignant renal tumors still depends on pathology. Percutaneous renal biopsy is the main preoperative examination. However, solid tumors show spatial and temporal inconsistencies in genetic and molecular pathways, microenvironment, tissues, and organs, limiting the accuracy and representativeness of biopsy results. With the innovation and development of medical imaging technologies, images can include the characteristics of tissue anatomy and physiological function. The advantages of non-invasive, comprehensive, and quantitative observation of imaging technology overcomes the shortcomings of biopsies, and can efficiently detect tumor heterogeneity [11, 12]. In 2012, Lambin et al. [11] proposed the concept of radiomics for the first time based on the heterogeneity of solid tumors. By extracting features from high-throughput image data, more reliable feature information can be extracted compared with that obtained from visual observation. Differential diagnosis between chRCC and RO is difficult by conventional diagnostic methods, and the use of radiomics for their differential diagnosis is rare. Accurate preoperative differentiation between chRCC and RO can aid better management of patients and help develop follow-up strategies. Additionally, it can mitigate the requirement and risk of radical nephrectomy in patients with RO. Therefore, in this study, we used a radiomics-based approach to analyze chRCC and RO and investigated the possibility of a higher preoperative diagnostic accuracy.

## Materials and methods

### Patients

A retrospective analysis of 44 cases of chRCC and 17 cases of RO was performed. The cases were confirmed pathologically in our hospital between 2008 and 2018, and the patients had undergone preoperative enhanced CT scan. There were 31 men and 13 women aged 22–79 years (average age 50.8 years) in the chRCC group and 9 men and 8 women aged 35–79 years (average age 54.9 years) in the RO group. Except for 8 cases without clinical data, most of the patients showed clinical signs that were non-specific (27/53) and then waist pain on the corresponding side or hematuria (20/53), and so on.

### CT examination

Most of the patients underwent multi-phase enhanced CT scanning, including a plain scan and phase scanning of the corticomedullary (CMP), nephrographic (NP), and excretory (EP) phases. In five of the 61 cases, the patients did not undergo EP scanning. Images for the 61 patients were captured using MDCT (LightSpeed VCT, GE Healthcare, Japan; SOMATOM Definition Flash, Siemens Healthcare, Germany) systems. The scanning ranged from the top of the diaphragm to the level of the iliac wing. The scanning parameters were tube voltage, 120 kV and scanning thickness, 5–8 mm. After the abdominal plain scan, a contrast agent was injected using a high-pressure syringe around the vein. The injection flow rate was 3 ml/s, and three-phase enhanced scanning was performed at 30–200 s.

### Tumor segmentation

**Fig. 1:**
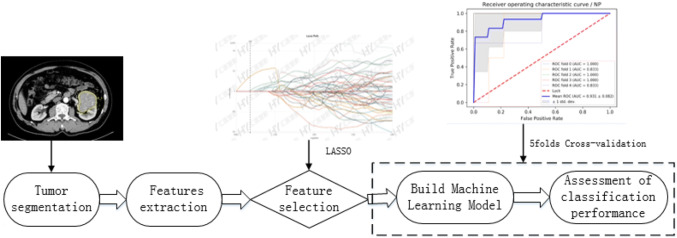
Basic flow chart showing the radiomics method devised for the differential diagnosis of renal chromophobe cell carcinoma and renal oncocytoma

The original digital imaging and communications in medicine image were imported into a post-processing platform (Big Data Intelligent Analysis Cloud Platform, Huiying Medical Technology Co., Ltd., Beijing). A radiologist manually delineated the region of interest (ROI) along the edge of the lesion, layer by layer, on each phase of the contrast-enhanced CT image. The volume of interest (VOI) of the lesion was automatically generated by the computer. Another senior radiologist examined the outline results. The criteria for delineation were as follows. CT axial images, except for the two planes where the lesion just appeared and was about to disappear, were evaluated. The ROI was used to delineate the boundary of all planes of the renal mass, including necrosis, cystic degeneration, and hemorrhage; however, it did not include the normal renal tissue or perirenal fat. Before extracting the VOI from the ROI, the window width and window position was adjusted to achieve the best contrast between the mass and the surrounding normal renal parenchyma. The window width and window positions were about 350 and 50 Hounsfield units (HU), respectively. Figure 1 shows the flow chart of the radiomics method.

### Feature extraction and selection

After delineating the VOI of each lesion, high-throughput data features based on feature classes and filter classes were automatically extracted from the aforementioned Radcloud platform. The features can be classified into three categories as follows: I. The characteristics of the intensity statistics, such as peak value, mean value, and variance, which are used to quantitatively describe the distribution of voxel intensity in CT images; II. Shape features, such as volume, surface area, and spherical value, which reflect the three-dimensional characteristics of the shape and size of the outlined area; and III. texture features, including the gray-level co-occurrence matrix, gray-level run length matrix, and gray-level size zone matrix, which can quantify the heterogeneity of the selected region. Additionally, Laplace–Gauss filtering, exponential, logarithmic, square, square root, and wavelet filters can be used to calculate the image intensity and texture features. Wavelet filters used included wavelet-LHL, wavelet-LHH, wavelet-HLL, wavelet-LLH, wavelet-HLH, wavelet-HHHH, wavelet-HHL, and wavelet-LLL.

First, all radiomic features were standardized using the StandardScaler function by Min–Max Scaling, and each set of feature values was mapped to the range of [0,1]. Then, a fivefold cross-validation was performed based on standardized features, and the optimal *λ* parameter was obtained from the minimum of the average mean square error by 1000 iterations. Finally, the least absolute shrinkage and selection operator (LASSO) feature selection algorithm was used to select the relevant features based on the optimal *λ* parameters, and the coefficients were calculated for each feature; then, radiomic features with non-zero coefficients were obtained. The LASSO algorithm can be used to reduce the dimensions of features and select the most meaningful features effectively [13, 14]. Further, using the *T* test on the optimum features between chRCC and RO patients, a probability value (*p* value) is calculated.

### Classifier training

Five classifiers, k-nearest neighbors (kNN), support vector machine (SVM), random forests (RF), logistic regression (LR), and multi-layer perception (MLP), were trained to construct the model by using fivefold cross-validation, which divided the data into five parts, training one part in turn, and estimating the accuracy of the algorithm by calculating the mean of the results of the five rounds of training. From these, the best model to distinguish chRCC from RO was selected. Finally, the performance of the feature classifier was validated and evaluated. The evaluation indicators included area under the curve (AUC), sensitivity, specificity and accuracy, accuracy, recall, and F1-score using the receiver operating characteristic curve (ROC).

## Results

### Feature selection of radiomics

**Fig. 2:**
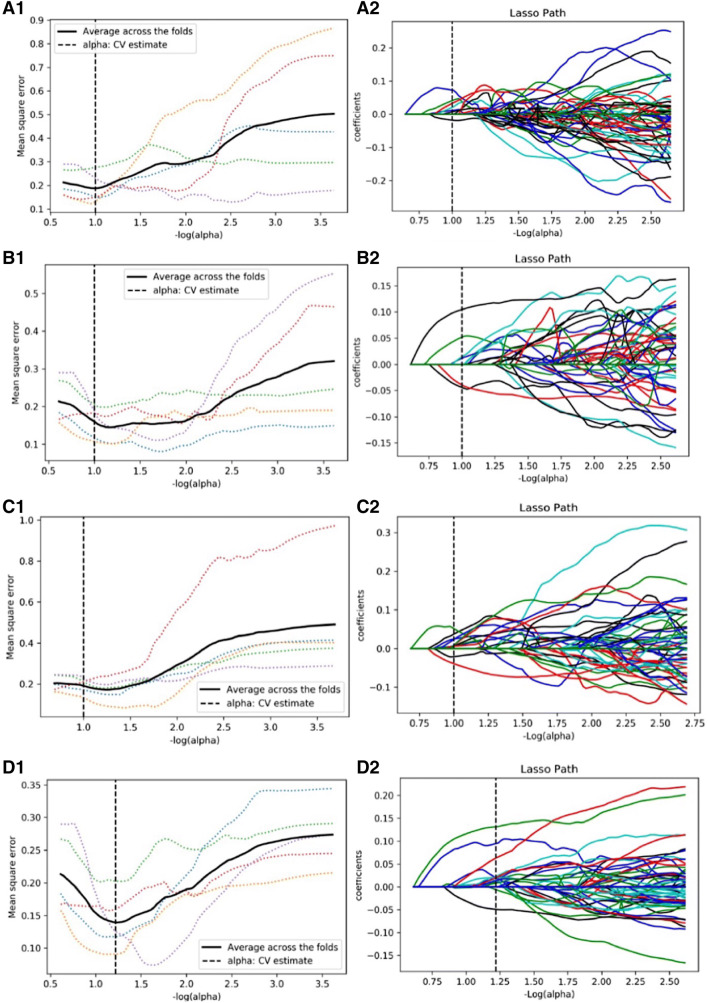
LASSO model on CMP (A1/A2), NP (B1/B2), and EP (C1/C2) images and a combination of CMP and NP (D1/D2) images. The optimal values of the LASSO tuning parameters were found (CMP: *λ* = 0.1 with Log(*λ*) = − 1; NP: *λ* = 0.1 with Log(*λ*) = − 1; EP: *λ* = 0.1 with Log(*λ*) = − 1; CMP and NP: *λ* = 0.063 with Log(*λ*) = − 1.2). And features which were correspond to the optimal alpha value were extracted following coefficients on images

A total of 1029 image features were extracted from each phase of enhanced images of each patient. The optimal *λ* parameter for the CMP, NP, and EP images features and a combination of CMP and NP images features (Fig. 2) were obtained. The LASSO algorithm was used to reduce the dimensionality of the above high-dimensional features based on the optimal *λ* parameters, and the best features were screened. These features were mainly texture and intensity statistical features, while only one morphological feature was screened out in the EP images. The combination of CMP and NP resulted in eight screened features from 2058 features, including five texture features and three intensity statistics features. The selected features and the corresponding *p* values are shown in Table 1. A radiomics set was built using the optimum features.

**Table 1:** Optimum features selected by the LASSO algorithm for enhancing high-dimensional features of each phase

Radiomic group	Radiomic feature	Associated filter	*p* value
CMP			
Texture features	Imc2	Original	< 0.0001
Firstorder	90Percentile	Square	< 0.0001
Firstorder	RobustMeanAbsoluteDeviation	Square	< 0.0001
Texture features	GrayLevelNonUniformityNormalized	Square	0.0131
NP			
Texture features	Imc1	Logarithm	0.0006
Firstorder	10Percentile	Square	0.0003
Texture features	SmallAreaLowGrayLevelEmphasis	Wavelet-HLH	< 0.0001
Firstorder	Mean	Wavelet-HLH	0.0036
EP			
Firstorder	10Percentile	Original	0.0004
Shape	Flatness	Original	0.0116
Texture features	Imc1	Logarithm	0.0088
Firstorder	10Percentile	Square	0.0004
Firstorder	Skewness	Wavelet-HLL	0.0312
Texture features	ClusterShade	Wavelet-HHH	0.0093
Combined CMP and NP			
Texture features	Imc2	Original	0.0004
Texture features	Correlation	Logarithm	0.0001
Firstorder	90Percentile	Square	< 0.0001
Firstorder	RobustMeanAbsoluteDeviation	Square	< 0.0001
Texture features	Imc1	Logarithm	0.0006
Texture features	SmallAreaEmphasis	Wavelet-LHH	0.0175
Texture features	SmallAreaLowGrayLevelEmphasis	Wavelet-HLH	< 0.0001
Firstorder	Mean	Wavelet-HHH	0.0036

*CMP* corticomedullary phase, *NP* nephrographic phase, *EP* excretory phase

*p* value < 0.05 indicates a significant difference in the Optimum features between chRCC and RO patients

### Diagnostic performance of radiomics models

**Fig. 3:**
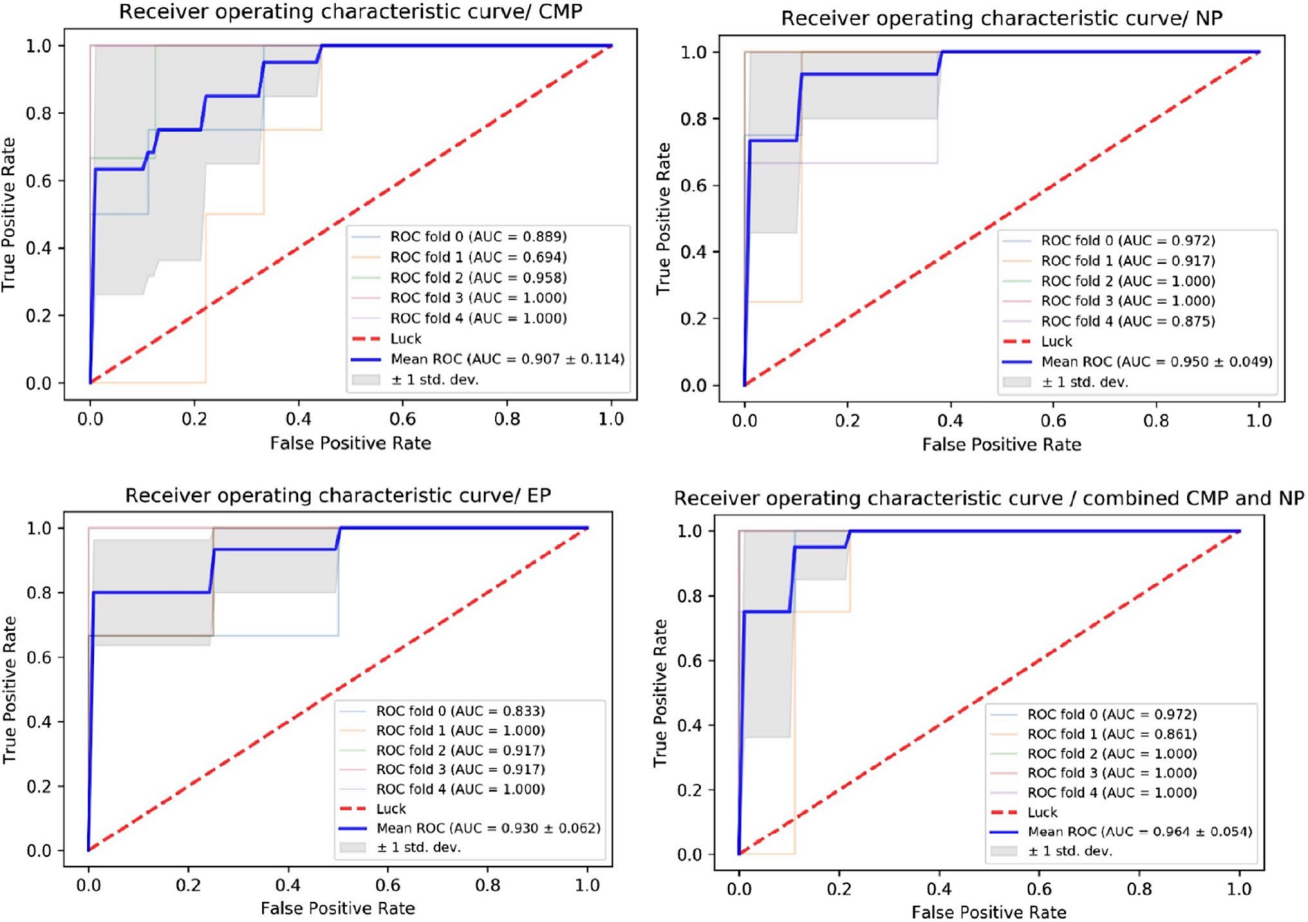
Receiver operating characteristic (ROC) curve for the support vector machine (SVM) classifier for the differential diagnosis of enhanced phase 3 (corticomedullary [CMP], nephrographic [NP], and excretory [EP] phases of contrast enhancement) and combined features of CMP and NP. *AUC* area under the curve

As shown in Table 2, AUC values under ROCs of multiple radiomics models obtained by a fivefold cross-validation method show that all models can obtain better diagnostic results, with AUC values greater than 0.850, and SVM being the best. The ROC curves of SVM at different enhancement stages are shown in Fig. 3. Four classifiers, kNN, SVM, LR, and MLP, used the combined features of CMP and NP to obtain the best discriminant diagnosis results for enhanced phase 3 (CMP, NP, and EP) and the combination of CMP and NP. Only the RF classifier obtained the best discriminant effect when analyzing features in NP phase. Table 3 compares the results of the five classifiers. The evaluation indexes included AUC sensitivity, specificity, accuracy, precision, recall, and F1-score.

**Table 2:** Average AUC for multiple histological models after fivefold cross-validation

Radiomic models	kNN	SVM	RF	LR	MLP
CMP	0.858 ± 0.180	0.907 ± 0.114	0.853 ± 0.146	0.915 ± 0.129	0.915 ± 0.129
NP	0.896 ± 0.097	0.950 ± 0.049	0.931 ± 0.082	0.942 ± 0.063	0.946 ± 0.081
EP	0.851 ± 0.130	0.930 ± 0.062	0.831 ± 0.087	0.831 ± 0.087	0.954 ± 0.046
Combined CMP and NP	0.925 ± 0.038	0.964 ± 0.054	0.910 ± 0.073	0.959 ± 0.065	0.959 ± 0.065

*AUC* area under the curve, *CMP* corticomedullary phase, *NP* nephrographic phase, *EP* excretory phase, *kNN* k-nearest neighbors, *SVM* support vector machine, *RF* random forests, *LR* logistic regression, *MLP* multi-layer perception

**Table 3:** Performance of the five feature classifiers for the differential diagnosis of chRCC and RO

	kNN	SVM	RF	LR	MLP
Sensitivity	0.952	0.999	0.929	0.881	0.929
Specificity	0.765	0.800	0.941	0.941	0.941
Accuracy	0.898	0.945	0.932	0.898	0.932
Precision	[0.909–0.867]	[0.930–1.0]	[0.975–0.842]	[0.974–0.762]	[0.975–0.842]
Recall	[0.952–0.765]	[1.0–0.8]	[0.929–0.941]	[0.881–0.941]	[0.929–0.941]
F-1 score	[0.930–0.812]	[0.964–0.889]	[0.951–0.889]	[0.925–0.842]	[0.951–0.889]

*chRCC* renal chromophobe cell carcinoma, *RO* renal oncocytoma, *kNN* k-nearest neighbors, *SVM* support vector machine, *RF* random forests, *LR* logistic regression, *MLP* multi-layer perception

## Discussion

In 2004, the World Health Organization formally classified chRCC as a new pathological classification of renal tumors. The incidence of chRCC is second only to that of renal clear cell and renal papillary cell carcinomas [3]; moreover, it has potential for metastasis. RO is a benign tumor with good prognosis [5]. Presently, surgical treatment, including partial and radical nephrectomy, is an effective method for treating local renal tumors. Radical nephrectomy can lead to an increased risk of chronic kidney disease, and is associated with an increased risk of cardiovascular disease morbidity and mortality. Compared to radical nephrectomy, a partial nephrectomy can preserve partial renal function, reduce overall mortality, and reduce the incidence of cardiovascular disease [2]. Therefore, radical nephrectomy should be avoided when nephron retention is achievable. Percutaneous renal biopsy is the most commonly used preoperative examination method, and it has 97% accuracy rate for distinguishing malignant renal masses [15]. However, the diagnosis of chRCC and RO on percutaneous renal biopsy presents difficulties [16].

chRCC and RO have many overlapping imaging features [17]. Clinically, it is difficult to distinguish chRCC from RO only by visual imaging. Currently, controversies exist on the imaging manifestations of the two kinds of tumors. For example, Rosenkrantz [10] stated that MRI features including fat, hemorrhage, margin of the mass, perirenal fat infiltration, renal vein cancer thrombus, enhancement uniformity, vascular proliferation and central scar, and segmental enhancement inversion cannot be used to distinguish between chRCC and RO. Wu et al. [9] reported that cases of RO present more instances of central scar, radial enhancement, and segmental enhancement inversion than those observed for chRCC on contrast-enhanced CT. Kim et al. [18] asserted that differential diagnosis between different tumor types can be achieved by CT enhancement. Most cases of chRCC showed homogeneous enhancement, whereas most renal clear cell and papillary cell carcinomas showed heterogeneous enhancement. Additionally, RO is characterized by homogeneous enhancement of the solid mass. The enhancement of RO and chRCC at each stage is lower than that of the normal renal cortex; however, enhancements of RO are more prominent than those of chRCC [9]. Thus, in clinical practice, it is difficult to distinguish chRCC from RO only by visual imaging. Therefore, this study used radiomics to differentiate chRCC from RO.

Texture analysis refers to the process of extracting texture feature parameters through certain image processing technologies, so as to obtain quantitative or qualitative description of texture. This technique can be used to detect subtle differences that cannot be detected by the naked eye and is more objective for tumor discrimination. Because chRCC and RO are relatively rare compared to renal clear cell and renal papillary cell carcinoma, radiomic studies of renal tumors are focused on relatively common renal tumors. Studies on the most frequently occurring renal clear cell carcinoma have focused on different aspects such as preoperative diagnosis [19–22], tumor grade [23], prognostic evaluation [24], and molecular analysis of the cancer genes [25–27]. Yu et al. [20] extracted the texture features of four types of renal tumors, including renal clear cell carcinoma, renal papillary cell carcinoma, chRCC, and RO. The tumors were classified by an SVM classifier, and the histogram feature median demonstrated an AUC of 0.882 for differentiating chRCC from RO. In this study, SVM was used to classify the features screened by CMP and NP. The AUC of differential diagnosis between chRCC and RO was found to be as high as 0.964, which is better than previously reported results. Zhang et al. [19] combined several texture features including SD, entropy, mean positive pixels, and kurtosis to differentiate renal clear cell carcinoma from non-transparent cell carcinoma. The value of the AUC was 0.94 ± 0.03 and the accuracy was 0.87 (sensitivity = 89%, specificity = 92%). Similar methods were used to differentiate between renal papillary cell carcinoma and chRCC, and the accuracy of differential diagnosis was 78%. Most of the subjects in the above study had renal malignant tumors, but no benign ROs were included in the comparison. Thus, this study has more clinical significance in terms of comparing chRCC with RO.

Considering the isodensity of tumor masses on plain CT scans, errors may easily occur while describing an ROI. Therefore, this study analyzed the contrast-enhanced images; however, we did not analyze the CT plain scan images. Some studies such as the one by Hodgdon et al. [21] only analyzed CT plain scan images of renal tumors. Using CT plain scan texture features and subjective visual features to differentiate and diagnose fat-deficient angiomyolipoma from other renal tumors, the accuracy of the classification methods based on texture features was found to be higher than that of radiologists’ subjective judgment of tumors or that observed with the use of an SVM classifier to identify renal tumors. The accuracy of differential diagnosis was about 83–91%. Schieda et al. [28] did not analyze the vascular characteristics of renal masses according to the nuclear grading system for chRCC; therefore, only CT plain scan images were used for radiomic analysis for clinical grading of the tumors. Importantly, to ensure the accuracy of the boundary delineation of the CT plain scans, it is still necessary to use the image of CT enhancement as a reference. Kocak et al. [23] analyzed the influence of different edge segmentation methods on feature selection and classification performance, including contour focusing and edge contraction by 2 mm. The results show that the latter method can extract more texture features; however, the former method has better reproducibility of features and better classification performance for the nuclear grading system-based classification of renal clear cell carcinoma. In this study, manual ROI extraction was performed to segment the edge contour of each transverse mass. Recently, a variety of mathematical techniques have been used in radiomics to quantify image textures, including statistical, Fourier, and wavelet analysis, and have been applied to the study of a variety of tumors. Varghese et al. [22] used multi-phase CT fast Fourier transform index to analyze the CT-enhanced solid and fat-deficient renal masses. Good classification results were obtained when distinguishing benign from the malignant renal masses, differentiating RO from chRCC, and RO from lipid-poor angiomyolipoma (AUC > 0.7).

Bektas et al. [29] used different machine learning classifiers, such as SVM, MLP, RF, kNN, and naive Bayes, for predicting Fuhrman nuclear grade of clear cell renal cell carcinomas (ccRCCs), and the best model was created using SVM (AUC = 0.851, accuracy = 0.913). Lee et al. [30] combined different feature selection methods and different feature classifiers, which included SVM, RF, kNN and LR, to distinguish benign fat-poor angiomyolipoma from malignant ccRCC. kNN and SVM classifiers with ReliefF feature selection achieved the best accuracy of 72.3 ± 4.6% and 72.1 ± 4.2%, respectively. The results of this study show that five classifiers have good diagnostic performance in feature classification methods (accuracy > 0.89, AUC > 0.90). One of the best models for the differential diagnosis between chRCC and RO was the use of SVM to classify the features screened by the combination of CMP and NP. The accuracy was found to be as high as 0.945, and the AUC was 0.964 ± 0.054. We suggest the use of radiomics of enhanced CT images for differentiating between chRCC and RO.

This study has some limitations. First, this was a single-center study and the sample size was small; notably, there were relatively fewer RO cases. A multi-center study of these rare cases must be undertaken under favorable conditions. Second, the CT equipment was not uniform and the scanning parameters were different, which may have influenced the repeatability of the results. Third, failure to analyze plain CT images may have led to the omission of internal masses. Considering the isodensity of tumor masses on plain CT scans, errors could have occurred when describing the ROIs for some characteristic information. To address this issue, only prominent enhancement phase images were analyzed.

In summary, we established a machine learning model that can distinguish chRCC from RO on enhanced CT images. These models are expected to help clinicians formulate better clinical diagnosis and devise improved treatment strategies. Our results indicate that radiomics can accelerate the development of personalized therapy.
